# Combined treatment using cross-leg free flap and the Masquelet technique: a report of two cases

**DOI:** 10.1080/23320885.2022.2039667

**Published:** 2022-04-04

**Authors:** Takeo Osaki, Yasuko Hasegawa, Ryosuke Tamura, Tomoaki Fukui, Keisuke Oe, Takahiro Niikura, Tadashi Nomura, Kazunobu Hashikawa, Hiroto Terashi

**Affiliations:** a Department of Plastic Surgery, Kobe University Graduate School of Medicine, Kobe, Hyogo, Japan; b Department of Orthopaedic Surgery, Kobe University Graduate School of Medicine, Kobe, Hyogo, Japan

**Keywords:** Masquelet technique, cross-leg free flap, osteomyelitis, reconstruction

## Abstract

We introduce a treatment that combines the cross-leg free flap with the Masquelet technique and describe two cases using this method for bone and soft tissue reconstruction. Both patients were successfully treated and ambulatory. This novel method can be safely performed using the delay technique, indocyanine-green angiography and near-infrared spectroscopy.

## Introduction

For the reconstruction of bone defects following lower extremity injuries, the Masquelet technique [1] as well as free bone flaps and distraction osteogenesis are performed. Masquelet bone reconstruction uses an induced membrane, which is formed around a polymethyl methacrylate cement that has been used to fill the bone defect. Advantages of this technique are less donor sacrifice, and that patients do not need to wear an external fixator for a prolonged period. In this method, a flap is required if there is a soft tissue defect, and a free flap is selected if a pedicle flap is difficult to perform. In cases where there are no suitable blood vessels near the defect, a vein graft or cross-leg flap may be considered. However, both methods are high risk. In a vein graft, if both artery and vein must be bypassed, the probability of thrombus formation increases. Even in the cross-leg flap, there is a possibility of flap necrosis following flap division because the flap is essentially on the bloodless cement. We report two such cases in which the Masquelet technique was completed successfully by performing a cross-leg free flap using the delay technique, indocyanine-green (ICG) angiography and near-infrared spectroscopy.

## Case report

### Surgical technique

**Figure 1.**
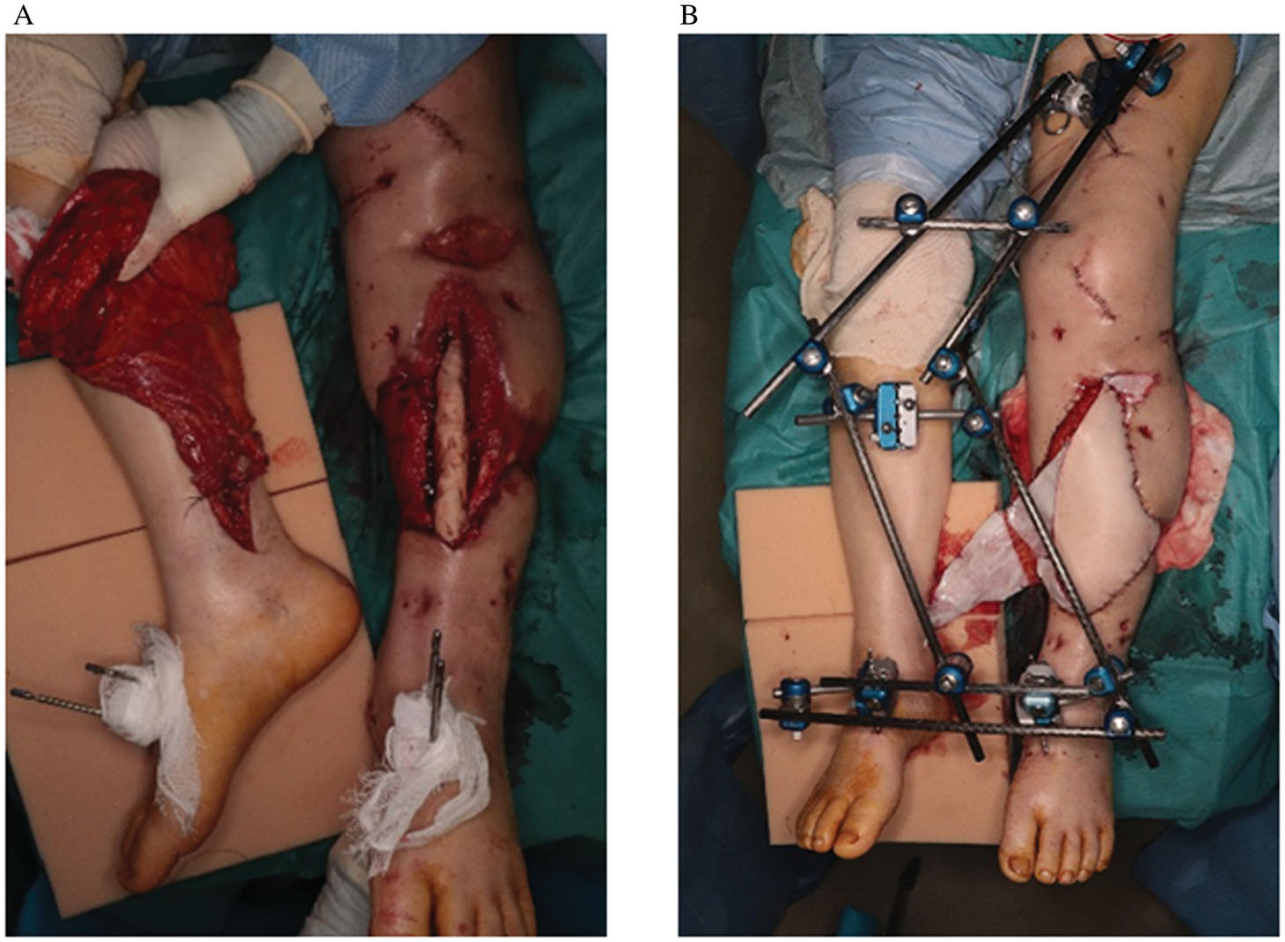
(A) Anastomosis of the latissimus dorsi (LD) flap to the blood vessel on the healthy side. (B) External fixation of both lower limbs.

**Figure 2.**
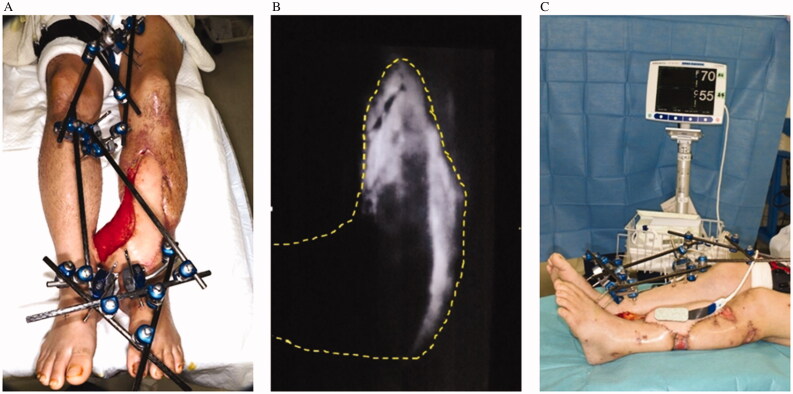
(A) Delay using a tourniquet on the femur of the healthy side. (B) Indocyanine-green angiography of the flap with blood circulation blocked from the pedicle on the healthy side. (C) The oxygen saturation of the flap is measured by near-infrared spectroscopy during delay with a tourniquet.

We report two cases of tibial osteomyelitis after injury with a soft tissue defect that required free tissue transplantation. The orthopedic surgeon debrided the infected sequestrum of the affected limb and performed cement placement. We evaluated the vasculature with CT angiography and applied this method in the absence of appropriate recipient vessels in the affected limb. In addition, the tissue blood circulation around the defect was examined using skin perfusion pressure or transcutaneous oxygen tension to see whether distant flaps were possible. Concurrent with the cement replacement, we transplanted the latissimus dorsi muscle flap with a flow-through type in the posterior tibial artery on the contralateral side. Both lower limbs were fixed using external fixation (Figure 1). After the operation, the delay was performed using a tourniquet on the femur of the healthy side or by clamping the flap pedicle once a week or more. At that time, we performed ICG angiography and near-infrared spectroscopy to confirm neovascularization from the affected limb to the flap (Figure 2). ICG angiography was mainly used to determine where blood circulation was poor in the flap. Near-infrared spectroscopy was applied to areas with poor blood circulation in the flap. Near-infrared spectroscopy was used to verify that the oxygen saturation of the flap did not decrease even when the flap pedicle was clamped, and the flap was then divided. Bone transplantation was performed in the subsequent weeks while awaiting the stabilization of the blood circulation of the flap after flap division.

### Case 1

**Figure 3.**
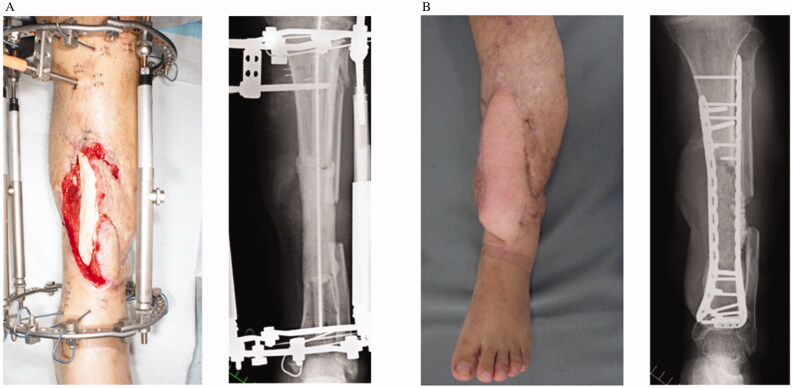
Case 1: (A) Preoperative clinical and radiographic findings. (B) Postoperative findings at 1 year.

A 42-year-old man presented with an open left tibia-fibula fracture and anterior tibial artery injury after a log hit his lower leg. He has no significant medical history. A previous physician had performed external fixation and repaired the anterior tibial artery. Subsequently, skin necrosis occurred, and a distally based sural flap and soleus muscle flap were performed. However, the bone was exposed due to muscle flap necrosis, and osteomyelitis was diagnosed based on radiological and wound culture findings; the patient was then transferred to our hospital three months after the initial trauma. The bone sequestrum was debrided, cement was placed, and external fixation was performed. A wide range of skin defects (approximately 15 × 7 cm) was found in the cement placement. The anterior tibial artery and the peroneal artery were occluded near the bifurcation, only the posterior tibial artery remained for supplying the scar tissue due to the multiple surgeries performed, making it unsuitable as a recipient of free flaps. To perform a free flap in the affected limb, a long vein graft had to be done in the scar, which was considered high risk. Therefore, simultaneously with cement replacement and plate fixation, a free latissimus dorsi muscle flap was transplanted to the left lower leg using the posterior tibialis artery of the right leg as the recipient. The flap was divided 51 days after the operation, and the flap survived. Five weeks after the flap was divided, autologous bone grafting with β-tricalcium phosphate granules (Affinos®, Kuraray, Co., Ltd., Tokyo, Japan) was performed (bone gap: 90 mm). Antibiotics were administered six weeks after bone sequestrum debridement and then perioperative only. One year after the operation, the transplanted bone radiologically healed, and the patient could walk independently with full weight-bearing without pain. Furthermore, no relapse of the infection and no ulcers or fistula formation were observed (Figure 3).

### Case 2

**Figure 4.**
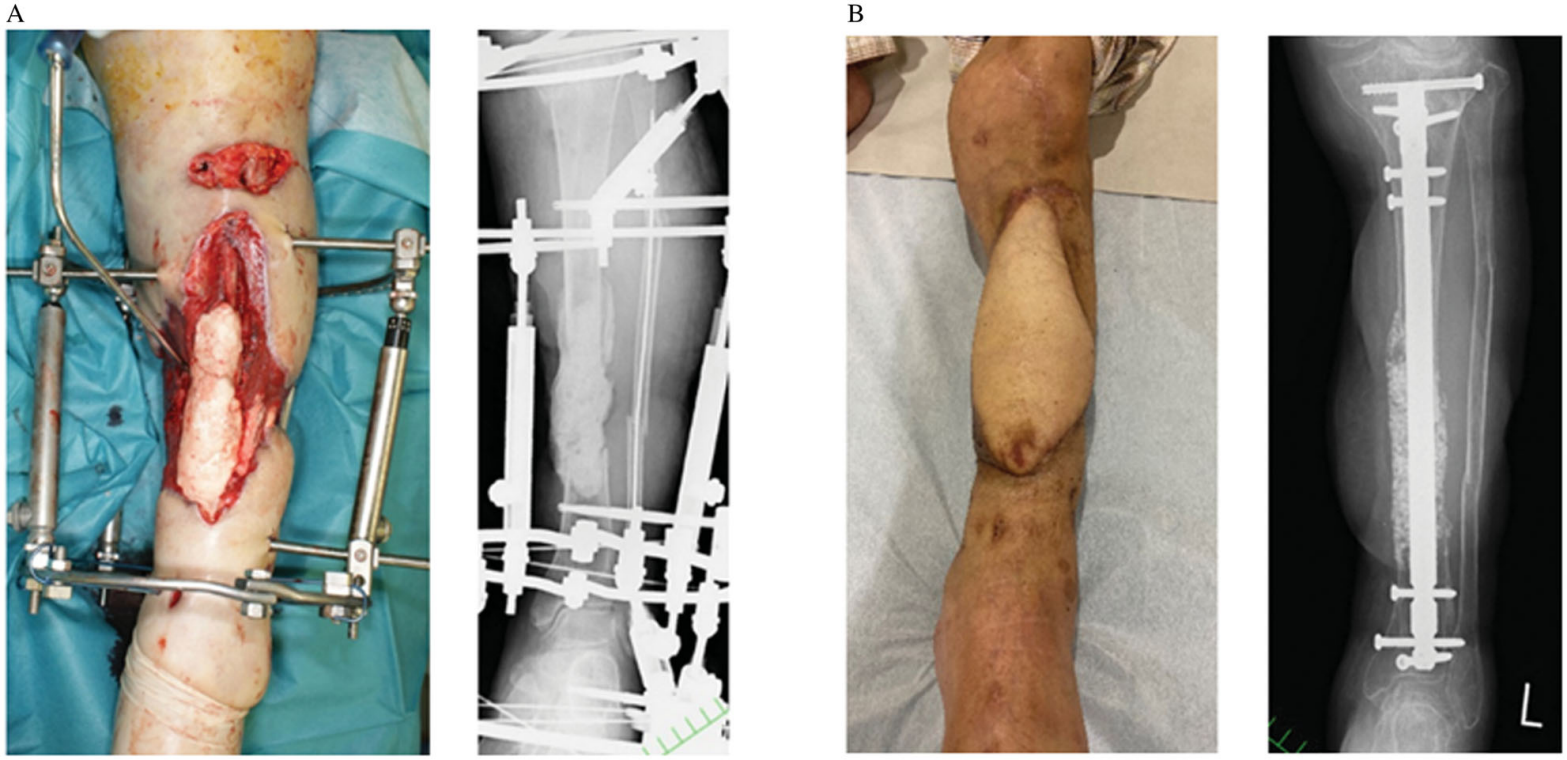
Case 2: (A) Preoperative clinical and radiographic findings. (B) Postoperative findings at six months.

A 72-year-old woman presented with an open left tibia-fibula fracture resulting from a car accident. Her medical history is significant for hypertension. Although a physician had previously treated her, skin necrosis was widespread, and infection had occurred. Therefore, she was transferred to our hospital about one month after the injury. At the time of transfer, her left lower leg had extensive skin and soft tissue defects, the length was 15 cm or more and the width was over half of the circumference, with extensive exposure of the bone. Bone in that area was diagnosed with osteomyelitis by culture examination and radiological findings. After bone debridement and cement placement, negative pressure wound therapy was performed with continuous irrigation until surgery. Both the anterior tibial artery and the peroneal artery were interrupted in the middle, and ultrasonography showed decreased blood flow in the posterior tibial artery. The saphenous vein was also occluded, and we determined that there was no suitable blood vessel for the recipient in the left lower limb. After the cement was replaced, a free latissimus dorsi myocutaneous flap was transplanted with the right posterior tibial artery as the recipient. The flap was divided 21 days after the operation, and the flap survived. Autologous bone grafting with Affinos^®^ granules was performed eight weeks after the flap division (bone gap: 80 mm). Six months after the operation, the transplanted bone was radiologically healed, and the patient could walk with a cane at full load. Oral antibiotics were taken for one year after the operation. There were no wounds or recurrence of infection (Figure 4).

## Discussion

The Masquelet technique involves two stages. In the first stage, debridement and cement spacers are placed in the bone defect to induce the formation of a membrane. In the second stage, the cement spacer is replaced with autologous cancellous bone. The membrane prevents the resorption of the graft and favors its vascularity and consolidation [2]. If soft tissue reconstruction is required, it is performed in the first stage. There are several reports on how to combine the Masquelet technique and flap [3–5]. Abdou et al. reported that by combining distraction osteogenesis or the Masquelet technique and flap, the bone union rate was 85% [5]. Hutson et al., reported healing in 18 of 19 fractures treated with Masquelet and flap, with a mean tibial bone defect of 9.4 cm [3]. Treatment with a combination of Masquelet technique and flap is therefore considered to be a useful method.

In the free flap surgery, if there are no suitable blood vessels around the soft tissue defect due to vascular damage, long vein graft is needed. However, this is considered to cause significant complications [6]. Therefore, as a method with a higher success rate, arteriovenous loops technique has been reported [7–9]. Arteriovenous loops have a constant high blood flow and facilitate vascular distention at physiological pressures, leading to good patency [10]. Although we have no experience, arteriovenous loops technique is considered a viable option in such cases.

On the other hand, the cross-leg free flap was introduced by Taylor in 1979 as a method of tissue transplantation using the blood vessels of the contralateral lower limb when there is no suitable blood vessel in the limb that requires soft tissue reconstruction [11]. Compared with the classical cross-leg pedicle flap, it has the advantage that extensive tissue reconstruction can be performed to avoid the donor morbidity of the healthy limb. There have been many reports of this method to date [12–18], and it is considered an established treatment method. To the best of our knowledge, although there is a report of using cross-leg pedicle flaps with the Masquelet technique [19], this is the first report combining cross-leg free flaps with the Masquelet technique.

When the cross-leg free flap is performed using the Masquelet technique, there is a high risk of ischemia of the flap following flap division because the flap is essentially on the bloodless cement. Therefore, we measured the skin perfusion pressure and transcutaneous oxygen tension of the affected limb before surgery to confirm blood circulation around the wound to enable flap engraftment. In addition, during surgery, the skin around the wound was denuded as much as possible to increase the adhesive area of the flap. We also consider the delay of the flap to be significant. The flap was divided after confirming neovascularization by ICG angiography, and tissue oxygen saturation was determined using near-infrared spectroscopy. ICG angiography has been described in previous studies for examining the extent of flap survival [20,21] and time for the forehead flap division [22,23]. It is considered a useful method for measuring flap survival. Near-infrared spectroscopy has been reported to be helpful as a monitor for free flaps [24].

There was a significant difference in the number of days until the flap could be divided between case 1 and 2 (51 days and 21 days respectively). In both cases, blood circulation in the flap was confirmed using ICG angiography and near-infrared spectroscopy, there was a difference in the time required for neovacularization of the flap. The reason is considered to be that the exposed area of cement was larger in case 1 than in case 2, and the blood circulation around the defect was poor due to multiple surgeries in case 1.

We believe that the cross-leg free flap can be safely performed with the Masquelet technique. This technique can be used to improve patient outcomes in cases that may otherwise have resulted in amputation of the affected limb owing to the absence of suitable recipient blood vessels.
